# CAIP-Induced ROS Production Contributes to Sustaining Atherosclerotic Process Associated with *Helicobacter cinaedi* Infection through Macrophages and Endothelial Cells Activation

**DOI:** 10.3390/ijms25179377

**Published:** 2024-08-29

**Authors:** Erika Paolini, Stefano Cozzi, Gaia Codolo

**Affiliations:** 1 Medicine and Metabolic Diseases, Fondazione IRCCS Cà Granda Ospedale Maggiore Policlinico, 20122 Milan, Italy; erika.paolini@policlinico.mi.it; 2 Department of Biology, University of Padova, 35131 Padova, Italy

**Keywords:** *Helicobacter cinaedi*, atherosclerosis, CAIP, LOX-1, ROS, oxidative stress, inflammation

## Abstract

Several lines of evidence have linked the intestinal bacterium *Helicobacter cinaedi* with the pathogenesis of atherosclerosis, identifying the Cinaedi Antigen Inflammatory Protein (CAIP) as a key virulence factor. Oxidative stress and inflammation are crucial in sustaining the atherosclerotic process and oxidized LDL (oxLDL) uptake. Primary human macrophages and endothelial cells were pre-incubated with 10 µM diphenyl iodonium salt (DPI) and stimulated with 20 µg/mL CAIP. Lectin-like oxLDL receptor (LOX-1) expression was evaluated by FACS analysis, reactive oxygen species (ROS) production was measured using the fluorescent probe H2DCF-DA, and cytokine release was quantified by ELISA assay. Foam cells formation was assessed by Oil Red-O staining, and phosphorylation of p38 and ERK1/2 MAP kinases and NF-κB pathway activation were determined by Western blot. This study demonstrated that CAIP triggered LOX-1 over-expression and increased ROS production in both macrophages and endothelial cells. Blocking ROS abrogated LOX-1 expression and reduced LDL uptake and foam cells formation. Additionally, CAIP-mediated pro-inflammatory cytokine release was significantly affected by ROS inhibition. The signaling pathway induced by CAIP-induced oxidative stress led to p38 MAP kinase phosphorylation and NF-κB activation. These findings elucidate the mechanism of action of CAIP, which heightens oxidative stress and contributes to the atherosclerotic process in *H. cinaedi*-infected patients.

## 1. Introduction

Cardiovascular diseases (CVDs) are considered the top cause of death in Western countries, with atherosclerosis being the most common cause of cardiovascular complications [1]. Atherosclerosis is a chronic inflammatory disorder of the medium and large arteries [2]. The accumulation of lipids in the arteries’ intima initiates the inflammation process, leading to a series of events that sustain the inflammatory atherosclerotic process [3,4].

Atherosclerosis is a multifactorial disease, and well-known risk factors include smoking, hypercholesterolemia, hypertension, and genetic predispositions [5]. However, in recent decades, there has been increasing interest in the hypothesis that infectious diseases may also contribute to the development of atherosclerosis by promoting chronic inflammation [6]. The association between specific microorganisms and the pathogenesis of atherosclerosis varies in terms of supporting evidence. While some infectious agents have been isolated from atherosclerotic plaques and demonstrated to exacerbate the disease in animal models, in other cases, only the DNA or antigens of microorganisms have been detected in atherosclerotic lesions [7]. Among the implicated microorganisms, *Chlamydophila pneumoniae* has garnered substantial evidence supporting its association with atherosclerosis [8,9]. Additionally, pathogens such as cytomegalovirus [10] and *Porphyromonas gingivalis* [11] have been reported to play a role in sustaining the inflammatory processes characteristic of atherosclerosis. Recently, attention has been drawn to *Helicobacter cinaedi* (Hc) after its isolation from an aortic aneurysm [12]. Hc antigens have been found in macrophages isolated from human atherosclerotic tissues [13,14,15], and the bacterium has been cultivated from aneurysms characterized by severe atherosclerosis and inflammation [16]. Hc is the most frequently non-gastric *Helicobacter* species isolated from humans, causing gastroenteritis or bloodstream infections [17]. Although it was initially described in immunocompromised patients, infections in immunocompetent patients have also been reported [18,19]. The asymptomatic nature of most Hc infections, except for fever, and the fastidious nature of this bacterial species make diagnosis challenging, contributing to the unknown prevalence of these infections in the general population [20,21]. For these reasons, the prevalence of the infections in the population is unknown. In comparison to other microorganisms implicated in atherosclerosis pathogenesis, Hc possesses the peculiar ability to invade the vascular system, causing endovascular infections, such as endocarditis and myopericarditis, and aneurisms [22]. Experimental evidence has demonstrated the etiological role of Hc in a hyperlipidemic mouse model of atherosclerosis [23].

The contribution of Hc to plaque formation is probably due to the promotion of pro-inflammatory cytokines within atherosclerotic lesions, as well as the activation of macrophages and their differentiation into foam cells [23,24]. In addition, it has been shown that the Hc-produced antigen CAIP plays a key role in atherosclerotic lesion progression by promoting the differentiation and maintenance of a pro-inflammatory profile in macrophages, leading to the expression of LDL receptors and subsequent intracellular lipid accumulation, thus contributing to foam cell formation [24].

However, the mechanisms underlying macrophage conversion into foam cells have not been fully elucidated. While previous observations indicated an increase in LDL receptor expression, responsible for the binding and internalization of unmodified LDL, this mechanism alone does not account for the entirety of LDL internalized by macrophages. Interestingly, the expression of scavenger receptors, such as CD36, responsible for binding oxLDL, does not increase upon CAIP stimulation [24]. Consequently, attention has been shifted to another scavenger receptor, LOX1, which in normal circumstances accounts for the 5–10% of ox-LDL uptake. However, in pro-inflammatory states, its expression is upregulated, contributing to the 40% of ox-LDL uptake [25,26].

The purpose of this study is to investigate the mechanisms through which CAIP may influence the expression of the LOX-1 receptor in macrophages and endothelial cells. In particular, given the critical role of ROS in the progression of atherosclerosis and their involvement in activating the signaling pathway leading to LOX-1 expression, we also examined the potential involvement of ROS in the pro-atherogenic effects mediated by CAIP. Our findings revealed a novel role for CAIP in triggering a milieu enriched with ROS, which in turn, contributes to the upregulation of LOX-1 expression.

## 2. Results

### 2.1. CAIP Increases the Expression of LOX-1 Receptor on the Surface of Macrophages and Endothelial Cells

**Figure 1:**
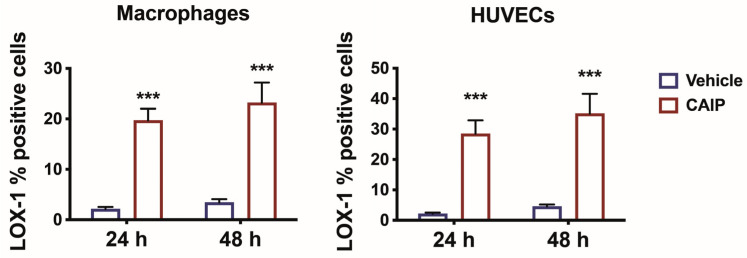
LOX-1 expression in macrophages and endothelial cells. Monocytes-derived macrophages and endothelial cells (HUVECs) were treated with CAIP 20 μg/mL or saline (vehicle). After 24 and 48 h, cells were harvested and analyzed by flow cytometry for the expression of LOX-1; cells were gated on Aqua negative cells (live cells). Data are expressed as mean % of positive cells ± SEM of three independent experiments from three different donors. Significance was determined by Student’s *t*-test: *** *p* < 0.001.

To investigate the involvement of the LOX-1 receptor in the pro-atherosclerotic inflammation induced by CAIP, we assessed its expression on primary human monocytes-derived macrophages (MDMs) and endothelial cells stimulated with the *H. cinaedi* antigen CAIP. Macrophages and human umbilical endothelial vein cells (HUVECs) were stimulated with CAIP for 24 and 48 h, after which the expression of the LOX-1 receptor was analyzed by flow cytometry. As depicted in Figure 1, both macrophages and HUVECs showed a significant increase in LOX-1 receptor expression following the CAIP stimulation at both time points, suggesting the involvement of the receptor in the pro-atherogenic effect mediated by CAIP.

CAIP increases reactive oxygen species (ROS) production in macrophages and endothelial cells.

**Figure 2:**
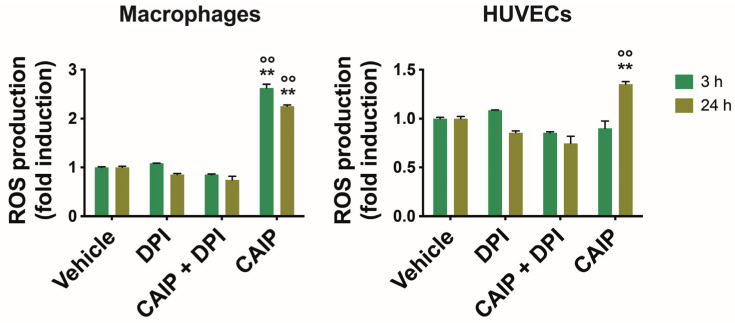
ROS production by macrophages and endothelial cells. Monocytes-derived macrophages and endothelial cells (HUVECs) were treated with CAIP 20 μg/mL or saline (vehicle); where indicated, cells were preincubated 1 h with 20 μM DPI. After 3 and 24 h, cells were incubated 30 min with H_2_DCFDA probe 10 μM and fluorescence analyzed with a fluorometer (Ex 485 nm, Em 535 nm). Data are expressed as n-fold vs vehicle ± SEM of three independent experiments from three different donors. Significance was determined by Student’s *t*-test: ** and °° *p* < 0.01 (** vs. vehicle, °° vs. CAIP + DPI).

In atherosclerosis, oxidative stress and the inflammatory process are strictly correlated, involving phagocytic cells and the endothelium. LDL reaching the sub-endothelial space undergoes oxidation (oxLDL) due to ROS generated by the oxidative stress, becoming oxidized. Macrophages are subsequently recruited to the vascular wall, where they encounter oxLDL through the LOX-1 receptor, transforming into foam cells. Vascular oxidative stress represents a pivotal aspect in the atherosclerotic process, with infectious agents or their antigens potentially exacerbating it, thereby acting as risk factors [27]. Recently, it has been observed that *H. cinaedi* is also capable of inducing the production of ROS through the NOX enzyme, thus promoting the formation of atherosclerotic plaques [23]. To investigate whether ROS are involved in the pro-atherogenic activity of CAIP, we assessed the CAIP capability to induce ROS production in macrophages and endothelial cells. An increase in ROS production was observed after 3 and 24 h in CAIP-treated macrophages, while in endothelial cells, ROS production was observed only after 24 h (Figure 2) [28].

To corroborate the link between CAIP and oxidative stress, we inhibited ROS production to ascertain whether the presence of the *H. cinaedi* antigen could amplify their production in macrophages and endothelial cells. Hence, cells were pre-incubated with DPI (diphenyleneiodonium chloride), a reversible inhibitor of the enzyme Nitric Oxide Synthase (NOS), expressed by both macrophages and endothelial cells, as well as other enzymes such as nicotinamide adenine dinucleotide phosphate oxidase (NADPH) oxidase or NADPH oxidase 2 (NOX2) [29]. As shown in Figure 2, the presence of DPI significantly reduced ROS production in macrophages and HUVEC cells stimulated with CAIP at 3 and 24 h.

### 2.2. The Heightened Expression of LOX-1 Is Facilitated by ROS Induced by CAIP

**Figure 3:**
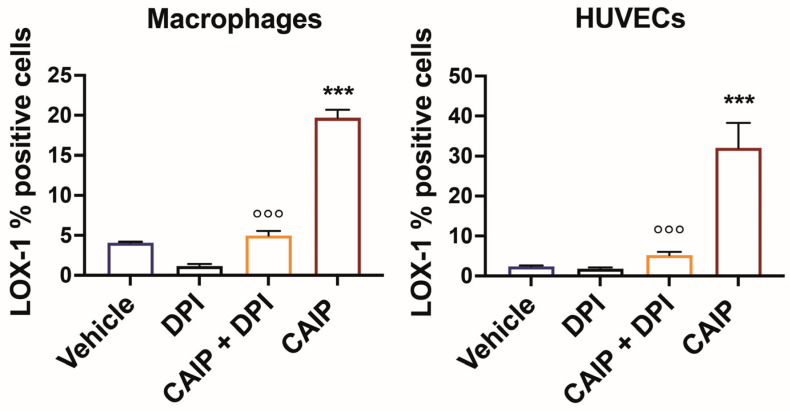
ROS inhibition affects CAIP-induced LOX-1 expression in macrophages and endothelial cells. Monocytes-derived macrophages and endothelial cells (HUVECs) were treated with CAIP 20 μg/mL or saline (vehicle); where indicated, cells were preincubated 1 h with 20 μM DPI. After 24 h, cells were harvested and analyzed by flow cytometry for the expression of LOX-1; cells were gated on Aqua negative cells (live cells). Data are expressed as mean % of positive cells ± SEM of three independent experiments from three different donors. Significance was determined by Student’s *t*-test: *** CAIP vs. Vehicle *p* < 0.001; °°° CAIP vs. CAIP + DPI *p* < 0.001.

Based on this evidence, it was investigated whether the effect of CAIP to promote increased LOX-1 receptor expression in macrophages and endothelial cells was mediated by ROS. To this end, macrophages and endothelial cells were pre-incubated with DPI, to inhibit ROS production, and subsequently stimulated or not with CAIP, followed by an assessment of LOX-1 expression through flow cytometry. Notably, the inhibition of ROS had a profound impact on reducing LOX-1 surface expression in both macrophages and endothelial cells (Figure 3) elicited by CAIP stimulation, strongly implying that CAIP-induced ROS play a pivotal role in the regulation of CAIP-mediated LOX-1 receptor expression.

### 2.3. CAIP-Mediated ROS Production Plays a Crucial Role in the Formation of Foam Cells

**Figure 4:**
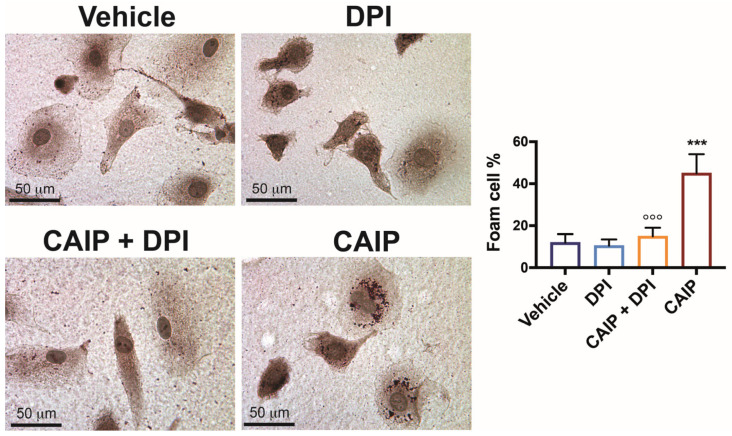
ROS are involved in CAIP-mediated foam cells formation. Monocytes-derived macrophages were exposed 24 h to CAIP or saline alone (vehicle) and preincubated, where indicated, 1 h with DPI. Cells were then fixed and stained with Oil Red O. Shown are representative images. Foam cells number was determined, and it is expressed as the percentage of total cells counted in 10 random fields. Foam cells quantification is expressed as mean value  ±  SD of three independent experiments, performed with three different cell preparations. Significance was determined by Student’s *t*-test between CAIP- versus vehicle-exposed cells (indicated by *) or vs CAIP + DPI-exposed cells (indicated by °). °°° *p* < 0.01, *** *p*  <  0.001.

Since the events described above are pivotal in atherosclerotic plaque development and destabilization, as they facilitate LDL oxidation and subsequent internalization by macrophages, leading to foam cell transformation [30], it was imperative to assess whether CAIP-induced ROS in macrophages indeed contribute to foam cell formation. To investigate this, macrophages were cultured on slides, pre-incubated with DPI, and then exposed to CAIP for 24 h. Following this incubation, lipid accumulation in the cells was determined using Oil Red O (ORO) staining. As shown in Figure 4, as already demonstrated previously [24], CAIP effectively promotes the conversion of macrophages into foam cells. In contrast, macrophages pre-incubated with DPI and subsequently stimulated with the *H. cinaedi* protein exhibit reduced lipid droplet accumulation, further affirming the involvement of ROS induced by CAIP in the process of foam cell formation.

### 2.4. The CAIP-Mediated Phosphorylation of MAPKs ERK1/2 and p38, and the Activation of NFkB, Are Dependent on ROS

It has been already shown that the activity of CAIP on macrophages involves a G protein-coupled receptor and the signaling pathways involving p38 and ERK [24]. Based on the evidence obtained in this study, we aimed to verify whether ROS induced by CAIP were responsible for the activation, and thus phosphorylation, of the p38 and ERK1/2 MAP kinases. Furthermore, it has been demonstrated that the expression of LOX-1 and oxidative stress are key events in the atherosclerotic process, as they lead to the activation of the transcription factor NF-κB, the ERK1/2 and p38 MAP kinases, and the enzyme NOX, events linked to endothelial dysfunction [27,31].

**Figure 5:**
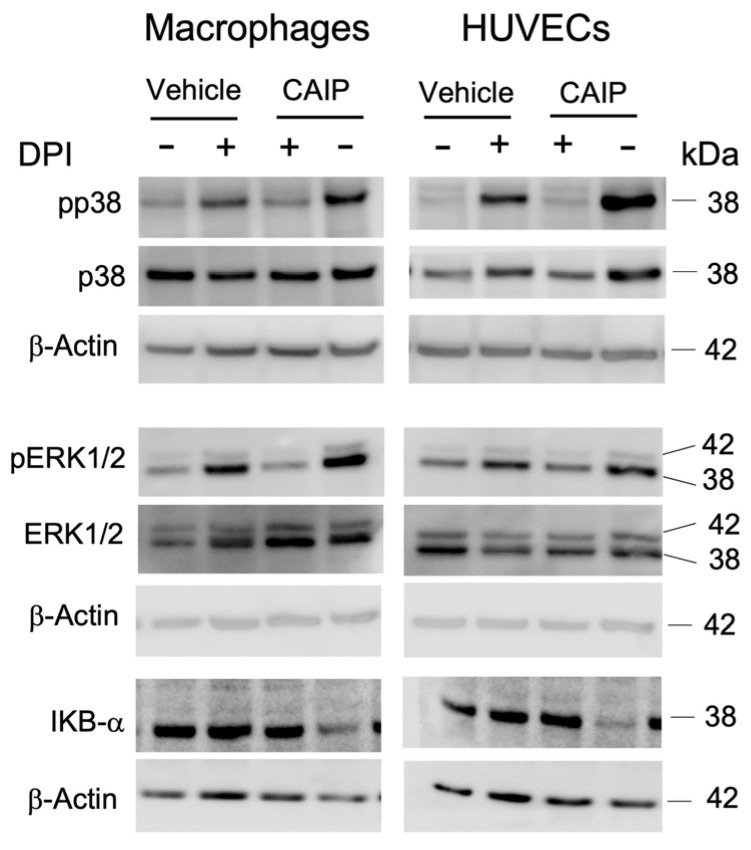
The signaling pathways activated by CAIP are mediated by ROS production. Monocytes-derived macrophages and endothelial cells (HUVECs) were treated with CAIP 20 μg/mL or saline (vehicle); where indicated, cells were preincubated 1 h with 20 μM DPI. After 1 h, cells were harvested and analyzed by Western blot. MAP kinases p38 and ERK1/2, their phosphorylated forms, and IKBα were revealed by specific antibodies. β-actin was used as the endogenous reference. Blot refers to a representative of two independent experiments, performed with cells from two different donors.

Macrophages and endothelial cells were pre-incubated with DPI and stimulated in the presence or absence of CAIP; after 1 h, the protein induced the phosphorylation of both kinases in macrophages alongside endothelial cells. In contrast, cells pre-incubated with DPI exhibited a reduced level of phosphorylation of both the p38 MAP kinase and ERK1/2 (Figure 5). This confirms that the ROS induced by the presence of the *H. cinaedi* protein are responsible for the phosphorylation of the p38 and ERK1/2 MAP kinases.

The binding of oxLDL to the LOX-1 receptor activates various signaling transduction mechanisms: the NADPH oxidase complex, the p38 and ERK1/2 MAP kinases, and the NF-κB transcription factor. The last is essential for the increased expression of adhesion molecules and chemokines, which facilitate the recruitment of monocytes to the site of infection. The production of ROS activates the NF-κB pathway, leading to an increase in the expression of LOX-1 and the consequent uptake of oxLDL, as well as pro-inflammatory cytokines and chemokines, which promote the adhesion of monocytes to endothelial cells [28]. Based on this evidence, we aimed to investigate whether the ROS produced by CAIP could mediate the activation of the NF-κB transcription factor. As depicted in Figure 5, it appears clear that after 1 h, CAIP induces IKBα degradation, thus promoting NF-kB activation, whereas in the presence of the ROS inhibitor, the CAIP-mediated IKBα degradation is reduced. This finding led to hypothesizing that, in this case as well, the ROS produced by CAIP are capable of mediating its activity by promoting a pro-inflammatory response. Therefore, oxidative stress, particularly the production of ROS by CAIP, should activate the NF-κB transcription factor, inducing the release of pro-inflammatory cytokines by macrophages present in the atherosclerotic plaque and increasing the expression levels of chemotactic molecules on the endothelial surface, thereby attracting immune cells to the site of infection.

### 2.5. CAIP-Triggered Cytokines Production Is Affected by ROS Inhibition

It is known that the binding of oxLDL to LOX-1 allows for the activation of the NADPH oxidase complex, which in turn, through the production of ROS, activates the transcription factor NF-κB, inducing the expression of genes encoding for adhesion molecules, cytokines, and chemokines, initiating the inflammatory response [26,30]. The ability of macrophages to induce the release of cytokines and chemokines in the presence of CAIP has been already demonstrated, which is capable of promoting the release of pro-inflammatory cytokines such as TNF-α, IL-23, IL-1β, and IL-6, as well as monocytes/macrophages recruiting chemokines CCL-2 and CCL-20 [24]. Furthermore, it has emerged that endothelial cells stimulated with CAIP are also capable of increasing the expression of adhesion molecules (VCAM-1 and E-selectin) and chemokines (CCL-2 and CCL-20), thereby recruiting mononuclear cells that contribute to the inflammatory response and promote atherosclerotic plaque instability [24].

**Figure 6:**
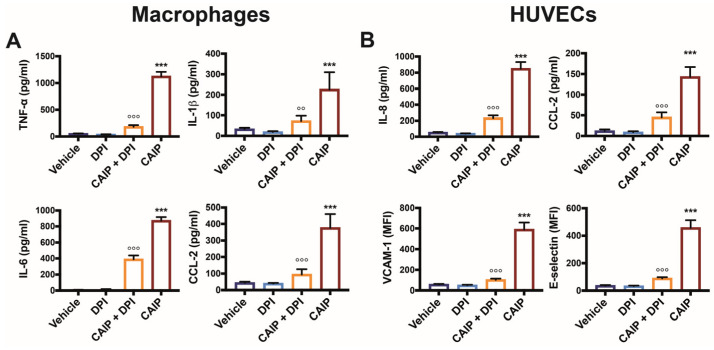
CAIP-mediated cytokines and adhesion molecules production by macrophages and endothelial cells is affected by ROS inhibition. Monocytes-derived macrophages and endothelial cells (HUVECs) were treated with CAIP 20 μg/mL or saline (vehicle); where indicated, cells were preincubated 1 h with 20 μM DPI. After 24 h, culture supernatants were collected and analyzed by ELISA assays, and cells were harvested and analyzed by flow cytometry. For TNF-a, IL-1b, IL-6, IL-8, and CCL-2 analysis, the cytokines content was determined by ELISA following the manufacturer’s instructions. Data are expressed as mean concentration values  ±  SEM of three independent experiments with cells derived from three different donors (for both macrophages and HUVECs). The surface expression of VCAM-1 and E-selectin was evaluated on cells gated on live cells. Data are expressed as Mean Fluorescence Intensiti (MFI) ± SEM of three independent experiments, performed with cells from three different donors. Significance was determined by Student’s *t*-test: *** CAIP vs. Vehicle *p* < 0.001; CAIP vs. CAIP + DPI °° *p* < 0.01, °°° *p* < 0.001.

Based on this evidence, we evaluated whether the production of ROS induced by CAIP was involved in the release of cytokines and chemokines by macrophages and endothelial cells. Figure 6 confirms that CAIP stimulation induces the production of TNF-α, IL-1β, IL-6, and CCL-2 in macrophages, as well as of IL-8 and CCL-2 in endothelial cells [24]. However, when oxygen radicals are inhibited with DPI, a significant reduction in the expression levels of cytokines and chemokines is observed in both macrophages and HUVEC endothelial cells stimulated with *H. cinaedi*’s protein.

Based on the data presented, it can be inferred that CAIP can induce the expression of pro-inflammatory cytokines and chemokines through ROS-dependent activation of the NF-κB factor.

## 3. Discussion

Multiple risk factors participate in the onset of atherosclerosis, encompassing both conventional factors such as hypertension, diabetes, and dyslipidemia, as well as non-traditional factors like oxidative stress and inflammation [32,33]. Oxidative stress, caused by an imbalance between ROS production and the effectiveness of antioxidant systems, has garnered significant attention [34]. It is now recognized as a pivotal element in every stage of the atherosclerotic process, from plaque formation to rupture. In addition to oxidative stress, inflammation is a fundamental driver of atherosclerosis, promoting the initiation and progression of atheromas and contributing to the onset of acute thrombotic events in unstable plaques [35]. These two processes, oxidative stress and inflammation, are closely connected and reinforce each other, accelerating plaque development and progression. In particular, LDL oxidation enhances the expression of adhesion molecules on endothelium, promoting the migration and infiltration of inflammatory cells into the vascular wall. Emerging evidence indicates that both vascular oxidative stress and inflammation can be triggered by infectious agents, which participate in atherosclerosis initiation by promoting endothelial dysfunction, LDL oxidation, and foam cell formation [36,37]. OxLDL activates MAPKs in vascular endothelial cells after endocytosis via the LOX-1, increasing the expression of MCP-1 and endothelial adhesion molecules. Furthermore, oxLDL promotes the subendothelial migration and differentiation of monocytes into macrophages and the foam cells formation; the interaction of OxLDL with LOX-1 also induces ROS production, and ROS themselves increase LOX-1 expression in cells, thus perpetrating the damage [27,38,39].

The findings of our study corroborate the current understanding of atherosclerosis pathogenesis. We demonstrated that the *Helicobacter cinaedi* antigen CAIP increases LOX-1 expression in both macrophages and endothelial cells. This aligns with prior research indicating that LOX-1 plays a pivotal role in oxLDL uptake and subsequent inflammatory responses. Notably, CAIP-induced LOX-1 expression was found to be ROS-dependent, as inhibition of ROS production significantly reduced LOX-1 expression.

This finding is consistent with previous studies showing that ROS play a crucial role in upregulating LOX-1 in pro-inflammatory states [27]. Accordingly, in physiological states, the LOX-1 expressed by macrophages contributes only to 5–10% of the oxLDL uptake; however, in pro-inflammatory conditions, the expression of LOX-1 is increased, contributing to 40% of the oxLDL update by macrophages [38,40]. Infections and pro-inflammatory cytokines amplify this phenomenon increasing the uptake of ox-LDL, thereby contributing to lipid uptake and foam cells formation. Furthermore, the oxLDL-LOX-1 pathway causes endothelial dysfunction, resulting in the increased expression of adhesion molecules and chemokines production [38,41].

Moreover, our results indicate that CAIP induces ROS production in macrophages and endothelial cells, further corroborating the role of ROS in atherosclerosis. The inhibition of ROS production using DPI markedly attenuated CAIP-induced ROS generation and subsequent LOX-1 expression. This suggests that ROS are critical mediators of CAIP’s pro-atherogenic effects, promoting oxidative stress and inflammatory responses within the vascular wall.

Our findings indicate that ROS are not only pivotal for LOX-1 expression but also for the subsequent formation of foam cells, a hallmark of atherosclerosis, driven by the uptake of oxLDL by macrophages. Macrophages exposed to CAIP and inhibited for ROS production showed a marked reduction in lipid accumulation, underscoring the importance of ROS in foam cell formation and, consequently, atherogenesis.

Since CAIP signaling involved the phosphorylation of ERK1/2 and p38 via the engagement of a G-coupled receptor [24], and oxidative stress in atherosclerosis associates with MAPKs activation [26], we investigated whether CAIP-associated ROS production was associated with MAPKs signaling in macrophages and endothelial cells. Our results demonstrate that, indeed, CAIP-induced phosphorylation of ERK1/2 and p38 MAPKs is dependent on ROS production. Additionally, we found that CAIP-induced NF-κB activation, a key transcription factor in inflammation, is mediated by ROS. This supports the notion that oxidative stress activates NF-κB signaling, promoting the expression of inflammatory cytokines and chemokines in atherosclerosis associated with *H. cinaedi* infection.

Overall, our study provides novel insights into the mechanisms through which *H. cinaedi* and its antigen CAIP contribute to atherosclerosis. The findings suggest that CAIP promotes atherosclerosis by inducing ROS production, which in turn upregulates LOX-1 expression, activates MAPK and NF-κB signaling pathways, and facilitates foam cell formation. These processes collectively contribute to the inflammatory and oxidative stress responses that drive atherosclerosis progression.

## 4. Materials and Methods

### 4.1. Ethics Statement

This investigation was conducted following ethical standards, the Declaration of Helsinki, and national and international guidelines. Umbilical cords, used for endothelial cells isolation, were obtained from full-term healthy pregnant women and were anonymously provided by the University Hospital of Padua, Italy. Informed consent was obtained from all subjects.

The human primary monocytes utilized in this study were isolated from buffy coats obtained from healthy blood donor volunteers and anonymously provided by the Transfusion Centre of the University Hospital of Padova, which concomitantly obtained the written informed consent for the use of the buffy coats for research purposes from blood donors. Data related to human samples were all analyzed anonymously.

Since buffy coats and umbilical cords were obtained not consequently to experimentation on human beings, but because of voluntary and informed donation, no approval of an ethics committee is needed in such cases in our institution.

### 4.2. Purification of the Cells and Treatments

Primary monocytes were prepared as described previously [42]. Briefly, peripheral blood mononuclear cells (PBMCs) from healthy donors were isolated by centrifugation on a gradient of Ficoll-Paque solution. Subsequently, cells were further separated on a gradient of Percoll 46% *v*/*v* solution in RPMI 1640 supplemented with 10% FCS. Cells were resuspended in RPMI containing 2% FCS and led to adhere to plastic wells for 1 h at 37 °C to further separate contaminating lymphocytes. Adherent monocytes were extensively washed with medium to remove residual non-adherent cells.

For macrophage differentiation, 5 × 10^5^ monocytes were seeded in 24-well plates and cultured in RPMI 20% FBS in the presence of 100 ng/mL M-CSF for 6 d. After 3 d, the medium was partially replaced. Once differentiated, macrophages were treated with 20 μg/mL CAIP or vehicle, in RPMI 1640 containing 10% FCS.

Human umbilical vein endothelial cells (HUVECs) were isolated from umbilical cords upon collagenase treatment and cultured as described elsewhere [43]. Briefly, the umbilical vein was cannulated and washed with sterile PBS; the vein was infused with 20 mL of collagenase 5 U/mL incubated 15 min at 37 °C. After washing with 30 mL of RPMI1640, 20% FBS, L-glutamine 2 mM, penicillin 100 U/mL, and streptomycin 100 μg/mL cells were harvested, centrifuged, and resuspended in complete medium (M199, 20% FBS, heparin 50 μg/mL, EGF 20 ng/mL, L-glutamine 2 mM, pen/strep) and grown in tissue culture plates (Costar, Washington, DC, USA) coated with 2% endotoxin-free gelatin. In addition, 4 × 10^4^ HUVECs were plated in 24-well plates and were exposed to 20 μg/mL CAIP or saline in M199 complete medium. Cells were used at passage 2–5.

When indicated, cells were pre-incubated with 20 μM diphenyleneiodonium chloride (DPI) (Merck, Rahway, NJ, USA) for 1 h before.

### 4.3. Flow Cytometry

For flow cytometric analysis, cells were detached with 5 mM Na-EDTA in PBS pH 7.5 and blocked with 10% HS in FACS buffer (PBS, 1% BSA) for 15 min at RT. Cells were stained with an anti-LOX-1 APC conjugated antibody (Biolegend, San Diego, CA, USA).

The fixable cell viability dye Aqua (Biolegend) was used to exclude dead cells from the analysis. Cells were washed and resuspended in FACS buffer and analyzed by BD LSFortessa X20 (Becton Dickinson, Franklin Lakes, NJ, USA). Values were expressed as the mean % of APC^+^ cells gated on live cells.

For evaluating the expression of adhesion molecules on HUVECs, cells were harvested and saturated as above. E-selectin (CD62E) and VCAM-1 (CD106) were revealed by anti-CD62E-FITC and CD106-PE (eBiosciences, ThermoFisher Scientific, Waltham, MA, USA), respectively. Values were expressed as the Mean Fluorescence Intensity (MFI) of VCAM-1 and E-selectin expressing cells, gated on live cells.

Data were analyzed using FlowJo version 10.3 (Tree Star Inc., Ashland, OR, USA).

### 4.4. ROS Detection

ROS production was measured as previously reported elsewhere [44]. Briefly, macrophages and HUVECs were treated with CAIP 20 μg/mL or saline as control and pre-incubated with 20 µM DPI where required. Cells were then incubated with 2′,7′-dichlorofluorescein diacetate (H2DCF-DA, 10 µM) in HBSS, glucose 10 mM for 45 min, and oxidant-sensitive probes. After washing, the fluorescence was measured with a microplate fluorometer (Ex at 480 nm and Em at 530 nm).

### 4.5. Foam Cell Formation

Foam cell formation was evaluated in macrophages treated with 20 μg/mL CAIP or saline and pre-incubated or not with 20 µM DPI, as previously reported [24]. After 24 h, cells were fixed in 4% buffered formalin for 10 min, washed in distilled water, rinsed in 60% isopropanol, and stained with 0.3% Oil Red O for 15 min. Cells were then cleaned in 60% isopropanol and counterstained with hematoxylin. Images were taken with a Leica DMR microscope with 63× magnification. Quantification of the lipid accumulation was performed with Fiji software (version 2.7), as reported elsewhere [45]: cells with more than 10 lipid droplets were defined as foam cells, and the percentage of foam cells formed in each condition was determined according to the previously described method.

### 4.6. Western Blot

Western blot was performed as reported elsewhere [46]. Briefly, cells were lysed in RIPA buffer (50 mM Tris–HCl, pH 7.4, 150 mM NaCl, NP-40 1%, Na-deoxycholate 0.5%, SDS 0.1%, 2 mM EDTA, 50 mM NaF, 1 mM Na3VO4, 1 mM EGTA, 2% PMSF) with 1 mM Protease Inhibitor Cocktails (PIC) and PhosSTOP (Merk, Darmstadt, Germany), and proteins were quantified by the BCA protein assay kit (ThermoFisher, Waltham, MA, USA), according to the manufacturer’s instructions. Twenty micrograms of proteins from each sample were separated electrophoretically in NuPAGE Bis-Tris 4–12% polyacrylamide gel (Novex, Life Technologies), and transferred on PVDF membranes (Merk). Membranes were blocked with 5% BSA (Sigma-Aldrich, St. Louis, MI, USA) in Tris-buffered saline (TBS, 50 mM Tris–HCl pH 7.6, 150 mM NaCl) containing 0.1% Tween20 (Sigma-Aldrich), and antigens were revealed using the following antibodies: mouse monoclonal antibody anti-ERK1/2 (1:2000, clone L34F12 Cell Signaling Technology, Danvers, MA, USA), anti-pERK1/2 monoclonal antibody (1:2000, clone E10 Cell Signaling Technology, Danvers, MA, USA), rabbit polyclonal anti-p38 (1:1000, Cell Signaling Technology), mouse anti-pp38 (1:2000, clone 28B10 Cell Signaling Technology), rabbit polyclonal anti-Ikbα antibody (1:1000 Cell Signaling Technology), and mouse anti-β-actin (1:10.000, Sigma-Aldrich). Blots were washed three times with TBS plus 0.1% Tween20 and incubated for 1 h at RT with horseradish peroxidase-conjugated anti-rabbit (Millipore, Burlington, MA, USA) or anti-mouse IgG secondary antibody (Novex, Life Technologies). Blots were developed with enhanced chemiluminescence substrate (EuroClone, Pero, MI, Italy), and the protein bands were detected using ImageQuant LAS 4000 (GE Healthcare Life Science, Chicago, IL, USA).

### 4.7. Detection of TNF-α, IL-1β, IL-6, CCL-2, and IL-8 in Culture Supernatants

Culture supernatants from macrophages and HUVECs were harvested and snap-frozen in liquid nitrogen until the cytokines/chemokines quantification, which was performed by ELISA assays, using specific kits (eBiosciences), following the manufacturer’s instructions.

### 4.8. Statistical Analysis

For statistical analysis, Student’s *t*-test was used; the differences were compared between the CAIP and Vehicle treatment and indicated by * in the figures, and the differences between CAIP and CAIP + DPI are indicated in the figures by °. Data are reported as the mean  ±  SEM.

## 5. Conclusions

In conclusion, our study highlights the significant role of *Helicobacter cinaedi* and its antigen CAIP in promoting atherosclerosis. Through inducing reactive oxygen species (ROS) production, upregulating LOX-1 expression, and activating MAPK and NF-κB signaling pathways, CAIP facilitates foam cell formation and drives the inflammatory and oxidative stress responses crucial to atherosclerosis progression. While our findings provide insights into the role of CAIP-induced oxidative stress in the atherosclerotic process associated with Hc infection, it is important to note that these results were obtained entirely from in vitro experiments. As such, further in vivo studies are necessary to validate these observations and fully understand the clinical relevance of CAIP in the context of atherosclerosis. Our findings, associated with in vivo studies, not only will deepen the understanding of the pathogenesis of atherosclerosis associated with *H. cinaedi* infection, but also pave the way for future research focused on developing targeted therapies against infectious agents and their antigens in cardiovascular diseases.

## Data Availability

Data is contained within the article.
